# Clinical and microbiological epidemiology of *Klebsiella pneumoniae* invasive disease in hospitalized adults in Johannesburg, South Africa: a multicentre observational study

**DOI:** 10.1093/jacamr/dlag003

**Published:** 2026-01-29

**Authors:** Denasha L Reddy, Ziyaad Dangor, Lyle Murray, Jacob Merika Tsitsi, Jeremy Nel, Trusha Nana, Jeannette Wadula, Rispah Chomba, Sinenhlanhla Ndzabandzaba, Vicky Baillie, Courtney P Olwagen, Shabir A Madhi

**Affiliations:** South African Medical Research Council Vaccines and Infectious Diseases Analytics Research Unit, Faculty of Health Sciences, University of the Witwatersrand, Chris Hani Road, Diepkloof, Soweto, Johannesburg 1864, South Africa; Department of Internal Medicine, Faculty of Health Sciences, University of the Witwatersrand, Johannesburg, South Africa; South African Medical Research Council Vaccines and Infectious Diseases Analytics Research Unit, Faculty of Health Sciences, University of the Witwatersrand, Chris Hani Road, Diepkloof, Soweto, Johannesburg 1864, South Africa; Department of Internal Medicine, Faculty of Health Sciences, University of the Witwatersrand, Johannesburg, South Africa; Division of Infectious Diseases, Department of Internal Medicine, Charlotte Maxeke Johannesburg Academic Hospital, Johannesburg, South Africa; Department of Internal Medicine, Faculty of Health Sciences, University of the Witwatersrand, Johannesburg, South Africa; Division of Infectious Diseases, Department of Internal Medicine, Chris Hani Baragwanath Academic Hospital, Johannesburg, South Africa; Department of Internal Medicine, Faculty of Health Sciences, University of the Witwatersrand, Johannesburg, South Africa; Division of Infectious Diseases, Department of Internal Medicine, Helen Joseph Hospital, Johannesburg, South Africa; Clinical Microbiology and Infectious Diseases, Faculty of Health Sciences, School of Pathology, University of the Witwatersrand, Johannesburg, South Africa; National Health Laboratory Service, Johannesburg, South Africa; Clinical Microbiology and Infectious Diseases, Faculty of Health Sciences, School of Pathology, University of the Witwatersrand, Johannesburg, South Africa; National Health Laboratory Service, Johannesburg, South Africa; Clinical Microbiology and Infectious Diseases, Faculty of Health Sciences, School of Pathology, University of the Witwatersrand, Johannesburg, South Africa; National Health Laboratory Service, Johannesburg, South Africa; Clinical Microbiology and Infectious Diseases, Faculty of Health Sciences, School of Pathology, University of the Witwatersrand, Johannesburg, South Africa; National Health Laboratory Service, Johannesburg, South Africa; South African Medical Research Council Vaccines and Infectious Diseases Analytics Research Unit, Faculty of Health Sciences, University of the Witwatersrand, Chris Hani Road, Diepkloof, Soweto, Johannesburg 1864, South Africa; South African Medical Research Council Vaccines and Infectious Diseases Analytics Research Unit, Faculty of Health Sciences, University of the Witwatersrand, Chris Hani Road, Diepkloof, Soweto, Johannesburg 1864, South Africa; South African Medical Research Council Vaccines and Infectious Diseases Analytics Research Unit, Faculty of Health Sciences, University of the Witwatersrand, Chris Hani Road, Diepkloof, Soweto, Johannesburg 1864, South Africa; Faculty of Health Sciences, Wits Infectious Diseases and Oncology Research Institute, University of the Witwatersrand, Johannesburg, South Africa

## Abstract

**Background:**

There is a paucity of information on the burden of *Klebsiella pneumoniae* invasive disease (KPn-ID) in Africa. We conducted a multicentre, observational study on the clinical and microbiological epidemiology of KPn-ID in hospitalized adults in South Africa, focusing on clinical outcomes and KPn susceptibility profiles.

**Methods:**

Surveillance for culture-confirmed KPn from blood and CSF was undertaken from 15 May 2023 to 14 May 2024. Phenotypic antimicrobial susceptibility was analysed, and the presence of carbapenemases was assessed with a lateral flow assay test.

**Results:**

We enrolled 524 of 617 individuals with KPn-ID. The median age was 48 (IQR: 35–61) years, and 84.4% (442/524) were presumed healthcare-associated infections. Comorbidities included HIV (26.9%; 141/524) and diabetes mellitus (16.4%; 86/524). There was a high prevalence of carbapenem resistance (55.0%; 288/524), with the OXA-48 carbapenemase detected in 71.5% (181/253), and OXA-48 and NDM co-detected in 20.9% (53/253) of tested isolates. Colistin resistance was detected in 7.6% (19/251) of tested isolates. The in-hospital case fatality risk (CFR) was 56.5% (296/524). Urethral catheterization [adjusted odds ratio (aOR) 3.30; 95% CI: 1.51–7.23] and an admission quick sepsis-related organ failure assessment score of 1 to 3 (aOR 2.14; 95% CI: 1.25–3.68) were independently associated with in-hospital death. Achieving source control was associated with lower odds of death (aOR 0.18; 95% CI: 0.10–0.30).

**Conclusions:**

We observed a high prevalence of MDR and high CFR in adults with KPn-ID. These data show the urgent need for strategies to mitigate KPn-ID in settings such as ours.

## Introduction

Approximately 1.27 million deaths were attributed to infections by bacteria exhibiting antimicrobial resistance (AMR) globally in 2019, with *Klebsiella pneumoniae* identified as the second leading pathogen, after *Escherichia coli*.^1^ Bloodstream infections (BSIs) due to *K. pneumoniae* associated with third-generation cephalosporin resistance from ESBL or AmpC-β-lactamase production, and carbapenem-resistant strains (CRKp) are associated with increased rates of treatment failure and high case fatality risk (CFR).^2^

*K. pneumoniae* with ESBL production or CRKp was estimated to cause 50 000 to 100 000 deaths globally in 2019,^1^ the majority of which occurred in Africa (∼50 000).^3^ Limited access to effective antimicrobials to manage CRKp may be contributing to the high burden of invasive *K. pneumoniae* deaths in Africa, where colistin is often the only available option for treatment of CRKp.^4^

Asymptomatic gastrointestinal colonization with *K. pneumoniae* is a major reservoir for the organism.^5^ Risk factors for CRKp gastrointestinal colonization include admission to an ICU, extensive invasive procedures, comorbidities (including diabetes, alcoholic liver disease, COPD, chronic kidney disease and malignancies) and carbapenem use.^5,6^ Gastrointestinal *K. pneumoniae* colonization may progress to invasive disease due to overgrowth of colonizing organisms and/or impairment of host immunity due to underlying immunosuppressive comorbidities or therapies.^2,5^

In high-income countries, the incidence of CRKp invasive disease is 1.3 per 10 000 hospital admissions, with a CFR up to 26.0%.^7^ There is a paucity of prospective studies on the burden of *K. pneumoniae* invasive disease (KPn-ID) among adults in Africa, or in regions with high prevalence of HIV infection. We conducted a multicentre, prospective, observational study to investigate the clinical and microbiological epidemiology of KPn-ID in hospitalized adults in Johannesburg, South Africa.

## Methods

### Study design and study population

South Africa has a population of 62,027,503, with Gauteng being the most populous and urbanized province, with an estimated population of 15 099 422.^8^ This observational study enrolled adults hospitalized with culture-confirmed KPn-ID across three academic hospitals in Johannesburg, South Africa: Chris Hani Baragwanath Academic Hospital (CHBAH), Charlotte Maxeke Johannesburg Academic Hospital (CMJAH) and Helen Joseph Hospital (HJH). The largest hospital, CHBAH, is located in Soweto and has a bed capacity of approximately 3200.^9^ CHBAH serves a population of over 1.9 million from southern Johannesburg. The second largest hospital, CMJAH, has a bed capacity of 1088, and serves the central Johannesburg region,^10^ and HJH is located in Auckland Park, Johannesburg and serves the population from Region B of the Johannesburg Municipality with a bed capacity of 636.^11^

The study was conducted from 15 May 2023 to 14 May 2024. Individuals with potential KPn-ID were identified through daily laboratory-based surveillance of blood and CSF cultures at the National Health Laboratory Service (NHLS) microbiology laboratories serving each hospital. Inclusion criteria included individuals ≥18 years of age who had *K. pneumoniae* cultured from either blood or CSF, thus meeting the study definition of invasive disease. Participants with multiple positive blood or CSF cultures during their admission were enrolled only once, on the basis of their first *K. pneumoniae* culture during the study period. The blood and CSF culture samples were collected at the discretion of the attending physicians, independent of the study. Participants were classified as presumed healthcare-associated infections (pHAIs) if the sample from which *K. pneumoniae* was cultured was obtained ≥48 h after admission to the hospital, or if there was evidence of previous contact with a healthcare service in the preceding 2 weeks. Presumed community-associated infections (pCAIs) were defined as *K. pneumoniae* cultured from a sample obtained <48 h after admission, and with no record of previous contact with a healthcare service in the preceding 2 weeks. Appropriate antibiotic treatment was defined as receipt of an antibiotic with appropriate tissue penetration to which the *K. pneumoniae* isolate was identified as being phenotypically susceptible on the final laboratory antimicrobial susceptibility report. The only exclusion criterion in the study was refusal of consent to participate.

### Microbiological identification and susceptibility testing

All participating NHLS microbiology laboratories were accredited by the South African National Accreditation System. Culture, identification and antimicrobial susceptibility testing of *K. pneumoniae* isolates were carried out at the NHLS laboratories according to standard operating procedures at each site. Briefly, *K. pneumoniae* was identified using either the API 20E (bioMérieux, France), the VITEK 2 automated system (bioMérieux, France) or the MALDI-TOF VITEK MS (bioMérieux, France) system. Antimicrobial susceptibility testing was performed using the VITEK 2 automated system or by disc diffusion, gradient diffusion or broth microdilution methods. Antimicrobial susceptibility results were interpreted following the 2023 and 2024 CLSI guidelines,^12,13^ except for tigecycline, for which the US FDA criteria were used. Isolates with intermediate susceptibility were differentiated from fully resistant isolates. Third-generation cephalosporin (3GC) resistance was based on phenotypic resistance to at least one of the three tested 3GCs (ceftriaxone, cefotaxime or ceftazidime). ESBL production was not specifically tested for in CRKp isolates, thus only 3GC resistance was reported in the study. Carbapenem resistance was based on phenotypic resistance to at least one of the three tested carbapenems (ertapenem, imipenem or meropenem). Colistin MIC testing was performed at NHLS laboratories using broth microdilution techniques on isolates with phenotypic carbapenem resistance. Additionally, carbapenemase detection was performed on isolates with phenotypic carbapenem resistance with the RESIST-5 O.K.N.V.I. lateral flow assay (Coris BioConcept, Belgium). MDR was defined as *K. pneumoniae* resistant to at least one agent in at least three antimicrobial categories.^14^

### Record review

Individuals with KPn-ID were approached to consent for study participation. Clinical and laboratory information was abstracted from clinical records and entered into a password-protected electronic database (REDCap). Clinical data included a history of comorbidities, including HIV coinfection and diabetes, and risk factors for disease such as recent surgery, central venous catheter and urethral catheterization, which were selected based on existing background literature. The presumed primary site of infection was based on clinical information (the diagnosis made by the attending clinician), radiological reports, and temporally related *K. pneumoniae* cultures from other sample types such as urine, pleural and peritoneal fluid, and vascular catheter tips. Information was abstracted regarding interventions performed to achieve source control of the infection. The Charlson Comorbidity Index (CCI) is a method of categorizing comorbidities based on the ICD by allocating an appropriate weight to each comorbidity based on the adjusted risk of mortality, with the sum of the weights resulting in a single comorbidity score for the patient. The CCI was calculated by abstracting information on comorbidities from the clinical records of all enrolled individuals. Participants were followed up until discharge from hospital or in-hospital death. A telephonic follow-up was done at 90 days after discharge to determine the outcomes of individuals who had been discharged from hospital.

### Statistical analysis

Continuous variables were described using medians and IQRs. Categorical variables were summarized using frequencies and percentages. The chi-squared test was used to compare two categorical variables. Logistic regression was used to assess potential risk factors for in-hospital death. Subsequently, adjusted odds ratios (aORs) and 95% CIs for in-hospital mortality were estimated using multivariate logistic regression analysis. The a priori variables included in the analysis as potential risk factors for death were selected due to their association with KPn-ID, as evidenced by existing background literature. The following variables were included a priori in the univariate analysis: area of admission during sample collection, the presence of comorbidities, recent surgery, the presence of a central venous line, the presence of a urethral catheter, category of infection, delay to receipt of appropriate therapy, polymicrobial culture, MDR, 3GC resistance, CRKp, oxacillinase-type β-lactamase (OXA-48), New Delhi metallo-β-lactamase (NDM), OXA-48 and NDM, and receipt of appropriate therapy. Variables included in the multivariate logistic regression were a priori risk factors for death, and variables in which the unadjusted *P* value was <0.1. Kaplan–Meier estimates of the survival function over the study period were generated, stratified by age category and pCAI or pHAI. Statistical analysis was performed using Stata/SE version 13.0 (StataCorp). A *P* value <0.05 was considered statistically significant.

### Ethical approval

The study was approved by the Human Research Ethics Committee (HREC), University of the Witwatersrand, and registered on the South African National Health Research Database (reference number: GP202209026). Informed consent was obtained for individuals enrolled during their hospital admission. If individuals were too ill to provide consent at the time of enrolment, proxy consent was obtained from their next-of-kin. A waiver for consent was granted by the HREC for cases where an individual died before consent could be obtained, to enable retrospective enrolment of the cases for whom medical records could be retrieved. Similarly, a waiver for consent was granted 3 months into the study to enrol individuals who were discharged before consent could be obtained.

## Results

**Figure 1.**
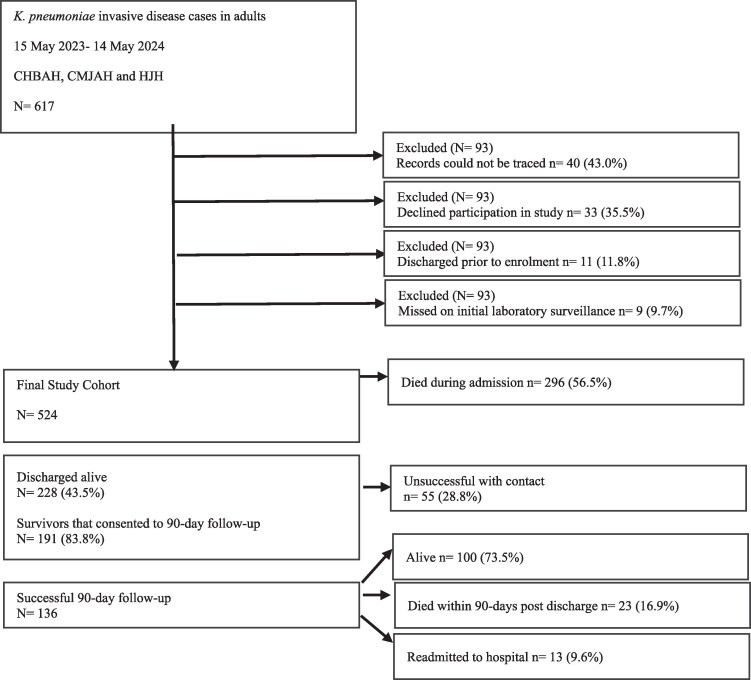
Study consort diagram.

Of the 617 individuals with KPn-ID identified over the study period, 524 (84.9%) were enrolled into the study (Figure 1), of whom 57.3% (300/524), 36.0% (189/524) and 6.7% (35/524) were from CHBAH, CMJAH and HJH, respectively. *K. pneumoniae* was cultured from blood and CSF in 98.9% (518/524) and 1.1% (6/524), respectively (Table S1, available as Supplementary data at *JAC-AMR* Online). Of the samples in which *K. pneumoniae* was cultured, 32% (168/524) were polymicrobial, with *Acinetobacter baumannii* (27.4%; 46/168), *Escherichia coli* (20.8%; 35/168) and *Enterococcus faecalis* (18.5%; 31/168) being the most common co-pathogens (Table S2).

Of the 524 enrolled individuals, 55.2% (*n* = 289) were male and 85.5% (*n* = 448) were black-African. The median age of participants was 48 (IQR: 35–61) years (Table 1). At the time when *K. pneumoniae* was cultured, most patients were admitted in the ICU (40.8%; *n* = 214), general medical wards (26.1%; *n* = 137) and surgical wards (18.5%; *n* = 97). It was noted that 17% (*n* = 90) of the cases had been re-hospitalized within 2 weeks of hospital discharge.

**Table 1.** Demographic and clinical characteristics of adults with *K. pneumoniae* invasive disease

Variable	Patients, *n* (%)^a^
Total cohort (*N* = 524)	
Male sex	289 (55.2)
Age, median [IQR], y	48 [35–61]
Age categories	
≥18–35 y	134 (25.6)
36–50 y	162 (30.9)
51–65 y	136 (26.0)
>65 y	92 (17.5)
Race	
Black African	448 (85.5)
White	36 (6.9)
Mixed race	31 (5.9)
Indian descent	9 (1.7)
Area of admission at time of sample collection	
ICU	214 (40.8)
High-care unit	12 (2.3)
Medical ward	137 (26.1)
Surgical ward	97 (18.5)
Oncology unit	29 (5.5)
Urology	10 (1.9)
Obstetrics and gynaecology	6 (1.2)
Transplant unit (solid organ)	6 (1.2)
Other^b^	13 (2.5)
Previous hospitalization within 14 d	90 (17.2)
Presence of comorbidities	
Yes	358 (68.3)
No	166 (31.7)
Charlson Comorbidity Index, median [IQR]	3 [1–6]
Comorbid conditions	
HIV/AIDS	141 (26.9)
Diabetes mellitus^c^	86 (16.4)
Solid tumour (non-metastatic)^d^	35 (6.7)
Moderate to severe chronic kidney disease	33 (6.3)
Cerebrovascular accident or transient ischaemic attack	26 (5.0)
Solid tumour (metastatic)^d^	25 (4.8)
Congestive cardiac failure	22 (4.2)
Leukaemia^d^	21 (4.0)
Lymphoma^d^	17 (3.2)
Liver disease^e^	17 (3.2)
Hemiplegia	16 (3.1)
COPD	15 (2.9)
Peripheral vascular disease	12 (2.3)
Peptic ulcer disease	12 (2.3)
Connective tissue disease	11 (2.1)
Dementia	10 (1.9)
Myocardial infarction	6 (1.1)
CD4+ T helper cells band (cells/mm^3^) in PLWH (*n* = 141)^a^	
<100	51 (36.2)
101–200	21 (14.9)
201–500	32 (22.7)
>500	21 (14.9)
Unknown/not recorded	16 (11.3)
Viral load band (copies/mL) in PLWH (*n* = 141)^a^	
≤50	52 (36.9)
51–999	19 (13.5)
1000–9999	6 (4.2)
≥10 000	40 (28.4)
Unknown/not recorded	24 (17.0)
ART (*n* = 141)^a^	
Not currently on ART	27 (19.2)
Tenofovir/lamivudine/dolutegravir	74 (52.5)
Abacavir/lamivudine/dolutegravir	31 (22.0)
Other regimen^f^	4 (2.8)
Unknown/not recorded	5 (3.5)
Recent surgery or intervention^g^	281 (53.6)
Presence of central venous catheter	266 (50.8)
Presence of urethral catheter	294 (56.1)

PLWH, people living with HIV.

^a^Data represent *n* (%) of patients unless otherwise specified.

^b^Other areas of admission include neurology (*n* = 5), otorhinolaryngology (*n* = 4), neurosurgery (*n* = 3), and maxillofacial surgery (*n* = 1).

^c^Diabetes mellitus was separated into uncomplicated disease (*n* = 30) and complicated disease with end-organ damage (*n* = 56).

^d^See Table S4 for details.

^e^Liver disease was separated into mild disease (*n* = 2) and moderate/severe disease (*n* = 15).

^f^Other ART regimens included zidovudine/lamivudine/lopinavir-ritonavir (*n* = 2), tenofovir/emtricitabine/lopinavir-ritonavir (*n* = 1) and zidovudine/lamivudine/dolutegravir (*n* = 1).

^g^See Table S5 for details.

Of the 524 KPn-ID cases, 68.3% (*n* = 358) had at least one comorbidity, the most common being HIV (26.9%; *n* = 141), diabetes mellitus (16.4%; *n* = 86) and non-metastatic cancer (6.7%; *n* = 35) (Table 1). The majority of KPn-ID cases living with HIV (PLWH) were receiving a dolutegravir-based antiretroviral regimen (74.5%; 105/141), whereas 19.2% (*n* = 27) were not receiving ART at the time of hospital admission. It was found that 37% (52/141) of PLWH had an HIV viral load <50 copies/mL, and 28.4% (*n* = 40) had an HIV viral load >10 000 copies/mL. Overall, 51.0% (72/141) of PLWH had CD4+ cell counts <200 cells/mm^3^. TB was the most common comorbid opportunistic infection diagnosed in PLWH with KPn-ID (17.7%; 25/141). The most common comorbid metastatic cancer was pancreatic carcinoma (1.1%; 6/524), whereas acute myeloid leukaemia was the most common cancer comorbidity overall (1.7%; 9/524). Other comorbid opportunistic infections associated with HIV, and a summary of cancer diagnoses, are detailed in Tables S3 and S4, respectively. Other underlying risk factors for KPn-ID included recent surgical procedures or interventions (53.6%; 281/524) (Table S5), presence of central venous catheter (50%; 266/524) or urethral catheter (56.1%; 294/524).

Of the 524 KPn-ID cases, 84.4% (*n* = 442) were categorized as pHAIs (Table 2). Overall, the probable primary sites of infection were respiratory (25.8%; *n* = 135), intra-abdominal (23.8%; *n* = 125) and urinary tract (16.8%; *n* = 88). When compared with pHAIs, pCAIs were more frequently associated with respiratory (39%; 32/82, *P* = 0.003) and urinary tract (26.8%; 22/82, *P* = 0.008) as the primary site of infection. Source control of the *K. pneumoniae* infection was achieved in 32.2% (169/524) of infections.

**Table 2.** Clinical presentation and outcomes in adults with *K. pneumoniae* invasive disease

Variable	Overall, *n* (%)^a^ (*N* = 524)	Presumed community-associated infections (*N* = 82)	Presumed healthcare-associated infections (*N* = 442)	*P* value^b^
Presumed primary site of infection^c^				
Respiratory tract	135 (25.8)	32 (39.0)	103 (23.3)	0.003
Intra-abdominal	125 (23.8)	14 (17.1)	111 (25.1)	0.117
Urinary tract	88 (16.8)	22 (26.8)	66 (14.9)	0.008
Skin and soft tissue	61 (11.6)	5 (6.1)	56 (12.7)	0.088
Vascular catheter	56 (10.7)	1 (1.2)	55 (12.4)	0.003
GI translocation neutropenia	26 (5.0)	2 (2.4)	24 (5.4)	0.252
Unknown	22 (4.2)	4 (4.9)	18 (4.1)	0.738
CNS	8 (1.5)	2 (2.4)	6 (1.4)	0.463
Bone and joints	3 (0.6)	0	3 (0.7)	ND
Source control of infection achieved				
Yes	169 (32.2)	28 (34.1)	141 (31.9)	0.689
No	263 (50.2)	38 (46.3)	225 (50.9)	0.448
Unknown	92 (17.6)	16 (19.5)	76 (17.2)	0.612
qSOFA score^d^				
0	280 (53.4)	36 (43.9)	244 (55.2)	0.060
1	173 (33.0)	31 (37.8)	142 (32.1)	0.315
2	55 (10.5)	7 (8.5)	48 (10.9)	0.528
3	15 (2.9)	8 (9.8)	7 (1.6)	<0.001
Unknown/not recorded	1 (0.2)	0	1 (0.2)	ND
Delay of ≥24 h until receipt of targeted therapy	322 (61.4)	18 (22.0)	304 (68.8)	<0.001
In-hospital outcome				
Died	296 (56.5)	34 (41.5)	262 (59.3)	0.003
Discharged^e^	228 (43.5)	48 (58.5)	180 (40.7)	0.003
Outcomes: successful 90 day follow-up (*n* = 136)^a^	(*n* = 136)^a^	(*n* = 23)^a^	(*n* = 113)^a^	
Alive	100 (73.5)	16 (69.6)	84 (74.3)	0.498
Died	23 (16.9)	5 (21.7)	18 (15.9)	0.411
Readmission to hospital	13 (9.6)	2 (8.7)	11 (9.7)	0.999

ND, not done; qSOFA, quick sepsis-related organ failure assessment.

^a^Data represent *n* (%) of patients.

^b^ *P* value resulting from chi-squared test comparing variables in presumed community-associated infection group with variables in presumed healthcare-associated infection group. The chi-squared test was not done where the value in one category was 0.

^c^Presumed primary site of infection based on clinical information (diagnosis made by treating clinician or ID physician and radiological investigations), supportive microbiological cultures from other sites.

^d^qSOFA score uses three criteria, assigning one point for low blood pressure (SBP ≥100 mmHg), high respiratory rate (≥22 breaths per minute), or altered mentation (Glasgow coma scale <15).

^e^Includes participants that consented to follow-up calls (*n* = 191) and those who did not consent to follow-up (*n* = 37).

Overall, phenotypic drug susceptibility testing of invasive *K. pneumoniae* isolates demonstrated 3GC resistance in 69.3% (363/524) of isolates, whereas 55.0% (288/524) of isolates were resistant to carbapenems (Table 3). Colistin resistance, with an MIC ≥4 mg/L, was detected in 7.6% (19/251) of isolates. Overall, 61.8% (324/524) of the *K. pneumoniae* isolates were MDR. An MDR phenotype was more common in isolates from pHAIs (72.9%, 322/442) compared with pCAIs (2.4%, 2/82; *P* < 0.001) (Table 3).

**Table 3.** Microbiological characteristics of *K. pneumoniae* invasive disease in adults

Variable	Overall, *n* (%)^a^ (*N* = 524)	Presumed community-associated infections (*N* = 82)	Presumed healthcare-associated infections (*N* = 442)	*P* value^b^
*Sample type*				
Blood culture	518 (98.9)	81 (98.8)	437 (98.9)	0.945
CSF	6 (1.1)	1 (1.2)	5 (1.1)	0.945
Polymicrobial bacteraemia	168 (32.1)	22 (26.8)	146 (33.0)	0.269
*Resistance^c^*				
Amoxicillin/clavulanic acid				
Resistant	362/521 (69.5)	9/81 (11.1)	353/440 (80.2)	<0.001
Intermediate	4/521 (0.8)	0/81 (0)	4/440 (0.9)	ND
Ciprofloxacin				
Resistant	299/510 (58.6)	6/81 (7.4)	293/429 (68.3)	<0.001
Intermediate	13/510 (2.5)	0/81 (0)	13/429 (3.0)	ND
Ceftriaxone				
Resistant	363/524 (69.3)	5/82 (6.1)	358/442 (81.0)	<0.001
Intermediate	0/524 (0)	0/82 (0)	0/442 (0)	ND
Ceftazidime				
Resistant	350/521 (67.2)	5/82 (6.1)	345/439 (78.6)	<0.001
Intermediate	0/521 (0)	0/82 (0)	0/439 (0)	ND
Cefepime				
Resistant	335/524 (63.9)	5/82 (6.1)	330/442 (74.7)	<0.001
Intermediate	0/524 (0)	0/82 (0)	0/442 (0)	ND
Gentamicin				
Resistant	239/521 (45.9)	3/82 (3.7)	236/439 (53.8)	<0.001
Intermediate	14/521 (2.7)	0/82 (0)	14/439 (3.2)	ND
Amikacin				
Resistant	173/523 (33.1)	0/82 (0)	173/441 (39.2)	ND
Intermediate	34/523 (6.5)	0/82 (0)	34/441 (7.7)	ND
Piperacillin/tazobactam				
Resistant	324/522 (62.1)	4/82 (4.9)	320/440 (72.7)	<0.001
Intermediate	9/522 (1.7)	0/82 (0)	9/440 (2.0)	ND
Ertapenem resistant				
MIC ≥2 mg/L	219/520 (42.1)	2/82 (2.4)	217/438 (49.5)	<0.001
MIC ≥1 and <2 mg/L	6/520 (1.2)	0/82 (0)	6/438 (1.4)	0.286
Resistant, no MIC result reported	40/520 (7.7)	0/82 (0)	40/438 (9.1)	ND
Imipenem resistant				
MIC ≥4 mg/L	172/522 (33.0)	1/82 (1.2)	171/440 (38.9)	<0.001
MIC ≥2 and <4 mg/L	23/522 (4.4)	1/82 (1.2)	22/440 (5.0)	0.070
Resistant, no MIC result reported	41/522 (7.9)	0/82 (0)	41/440 (9.2)	ND
Meropenem resistant				
MIC ≥4 mg/L	187/521 (35.9)	1/82 (1.2)	186/439 (42.4)	<0.001
MIC ≥2 and <4 mg/L	21/521 (4.0)	0/82 (0)	21/439 (4.8)	0.038
Resistant, no MIC result reported	34/521 (6.5)	0/82 (0)	34/439 (7.7)	ND
Tigecycline	24/223 (10.8)	0/19 (0)	24/204 (11.8)	ND
Colistin^d^				
MIC ≤2 mg/L	232/251 (92.4)	1/1 (100.0)	231/250 (92.4)	ND
MIC ≥4 mg/L	19/251 (7.6)	0/1 (0)	19/250 (7.6)	ND
Ceftazidime/avibactam	1/4 (25.0)	0 (0)	1/4 (25.0)	ND
Resistance pattern				
3GC^e^	363/524 (69.3)	5/82 (6.1)	358/442 (81.0)	<0.001
CRKp^f^	288/524 (55.0)	2/82 (2.4)	286/442 (64.7)	<0.001
MDR^g^	324/524 (61.8)	2/82 (2.4)	322/442 (72.9)	<0.001
Carbapenemase tested^h^				
OXA-48	181/253 (71.5)	2/2 (100.0)	179/251 (71.3)	<0.001
NDM	17/253 (6.7)	0/2 (0)	17/251 (6.8)	0.071
OXA-48 and NDM	53/253 (20.9)	0/2 (0)	53/251 (21.1)	0.001
Carbapenemase not detected	2/253 (0.8)	0/2 (0)	2/251 (0.8)	ND

CRKp, carbapenem-resistant *K. pneumoniae*; ND, not done; NDM, New Delhi metallo-β-lactamase; OXA-48, oxacillinase-type β-lactamase; 3GC, third-generation cephalosporin.

^a^Data represent *n* (%) of patients.

^b^ *P* value resulting from chi-squared test comparing variables in presumed community-associated infection group with variables in presumed healthcare-associated infection group. The chi-squared test was not done where the value in one category was 0.

^c^Resistance reported as fully resistant and intermediate resistant.

^d^Colistin MIC testing was performed at NHLS laboratories using broth microdilution techniques on isolates with phenotypic carbapenem resistance.

^e^Third-generation cephalosporin resistance was confirmed by resistance to either ceftriaxone, cefotaxime or ceftazidime.

^f^Carbapenem-resistant *K. pneumoniae* (CRKp) refers to strains of *K. pneumoniae* that inactivate carbapenems, either through the action of carbapenemase enzymes or other mechanisms, such as loss of outer membrane proteins or activation of efflux pumps.

^g^MDR was defined as *K. pneumoniae* resistant to ≥3 antimicrobial categories.

^h^Carbapenemase detection was performed on isolates with phenotypic carbapenem resistance with the RESIST-5 O.K.N.V.I. lateral flow assay (Coris BioConcept, Belgium).

Phenotypic carbapenemase production was tested in 87.8% (253/288) of CRKp isolates, of which 20% (53/253) had both OXA-48 and NDM carbapenemases detected, whereas OXA-48 was exclusively detected in 71.5% (181/253) and NDM exclusively detected in 6.7% (17/253). No carbapenemase was detected in 0.8% (2/253) of CRKp isolates tested. Two pCAI isolates (0.8%; 2/253) harboured a carbapenemase, OXA-48, with the majority of OXA-48-positive isolates being pHAI (70.8%; 179/253, *P* < 0.001).

Of the 524 KPn-ID cases, 61.5% (*n* = 322) experienced a delay of at least 24 h between sample collection for bacterial culture and the receipt of appropriate antibiotic therapy, more so in pHAI (68.8%; 304/442) than pCAI cases (22%; 18/82, *P* < 0.001) (Tables 2 and 4). Overall, 65.1% (341/524) of individuals received an antibiotic to which the *K. pneumoniae* was susceptible, including treatment with piperacillin/tazobactam (33.0%; *n* = 173), amoxicillin/clavulanic acid (31.7%; *n* = 166), and meropenem (30.0%; *n* = 157) (Table 4 and Figure S1). Colistin was prescribed to 24.2% (127/524) of individuals with KPn-ID, whereas tigecycline was prescribed to 4.2% (22/524) and ceftazidime/avibactam to 1.0% (5/524).

**Table 4.** Antibiotics received in adults with *K. pneumoniae* invasive disease

Variable	Overall, *n* (%)^a^ (*N* = 524)	Presumed community-associated infections (*N* = 82)	Presumed healthcare-associated infections (*N* = 442)
Received appropriate antibiotic^b^	341 (65.1)	71 (86.6)	270 (61.1)
Did not receive appropriate therapy^c^	179 (34.2)	10 (12.2)	169 (38.2)
Unknown/not recorded	4 (0.8)	1 (1.2)	3 (0.7)
Time from sample collection until receipt of appropriate antibiotic	*N* = 341	*N* = 71	*N* = 270
Already on appropriate therapy	59 (17.3)	24 (33.8)	35 (13.0)
<24 h	91 (26.7)	35 (49.3)	56 (20.7)
24 h	39 (11.4)	4 (5.6)	35 (13.0)
48 h	41 (12.0)	4 (5.6)	37 (13.7)
72 h	38 (11.1)	3 (4.2)	35 (13.0)
>72 h	64 (18.8)	1 (1.4)	63 (23.3)
Unknown/not recorded	9 (2.6)	0	9 (3.3)
Received appropriate antibiotic	*N* = 341	*N* = 71	*N* = 270
3GC susceptible	142 (41.6)	69 (97.2)	73 (27.0)
3GC resistant	199 (58.4)	2 (2.8)	197 (73.0)
CRKp	141 (41.3)	0	141 (52.2)
MDR	168 (49.3)	0	168 (62.2)
Antibiotic received			
Amoxicillin/clavulanic acid	166 (31.7)	51 (62.2)	115 (26.0)
Ciprofloxacin	24 (4.6)	4 (4.9)	20 (4.5)
Ceftriaxone	74 (14.1)	23 (28.0)	51 (11.5)
Ceftazidime	10 (1.9)	0	10 (2.3)
Cefepime	33 (6.3)	4 (4.9)	29 (6.6)
Gentamicin	14 (2.7)	2 (2.4)	12 (2.7)
Amikacin	144 (27.5)	5 (6.1)	139 (31.4)
Piperacillin/tazobactam	173 (33.0)	15 (18.3)	158 (35.7)
Ertapenem	93 (17.7)	6 (7.3)	87 (19.7)
Imipenem	110 (21.0)	6 (7.3)	104 (23.5)
Meropenem	157 (30.0)	3 (3.7)	154 (34.8)
Colistin	127 (24.2)	1 (1.2)	126 (28.5)
Tigecycline	22 (4.2)	0	22 (5.0)
Ceftazidime/avibactam	5 (1.0)	0	5 (1.1)

3GC, third-generation cephalosporin; CRKp, carbapenem-resistant *K. pneumoniae.*

^a^Data represent *n* (%) of patients.

^b^Appropriate antibiotic treatment was defined as the receipt of an antibiotic with appropriate tissue penetration to which the *K. pneumoniae* isolate was found to be susceptible on the final antimicrobial susceptibility report.

^c^Patients who did not receive appropriate therapy are described further in Table S6.

Overall, 34.2% (179/524) of individuals did not receive appropriate antibiotic therapy (appropriate antibiotic therapy refers to receipt of active therapy, as defined under ‘Methods’ above), including 2.5% (13/524) who were not started on any antibiotic and 31.7% (166/524) who did not receive targeted antibiotic therapy (Table S6). The most frequent reason for not receiving any antibiotic was death on the day of culture prior to antibiotic administration (53.8%; 7/13). The most frequent reasons for not receiving targeted therapy were failure of the clinician to act on the drug susceptibility result (38.0%; 63/166) and death on the day of culture prior to the availability of the drug susceptibility result (25.9%; 43/166).

**Figure 2.**
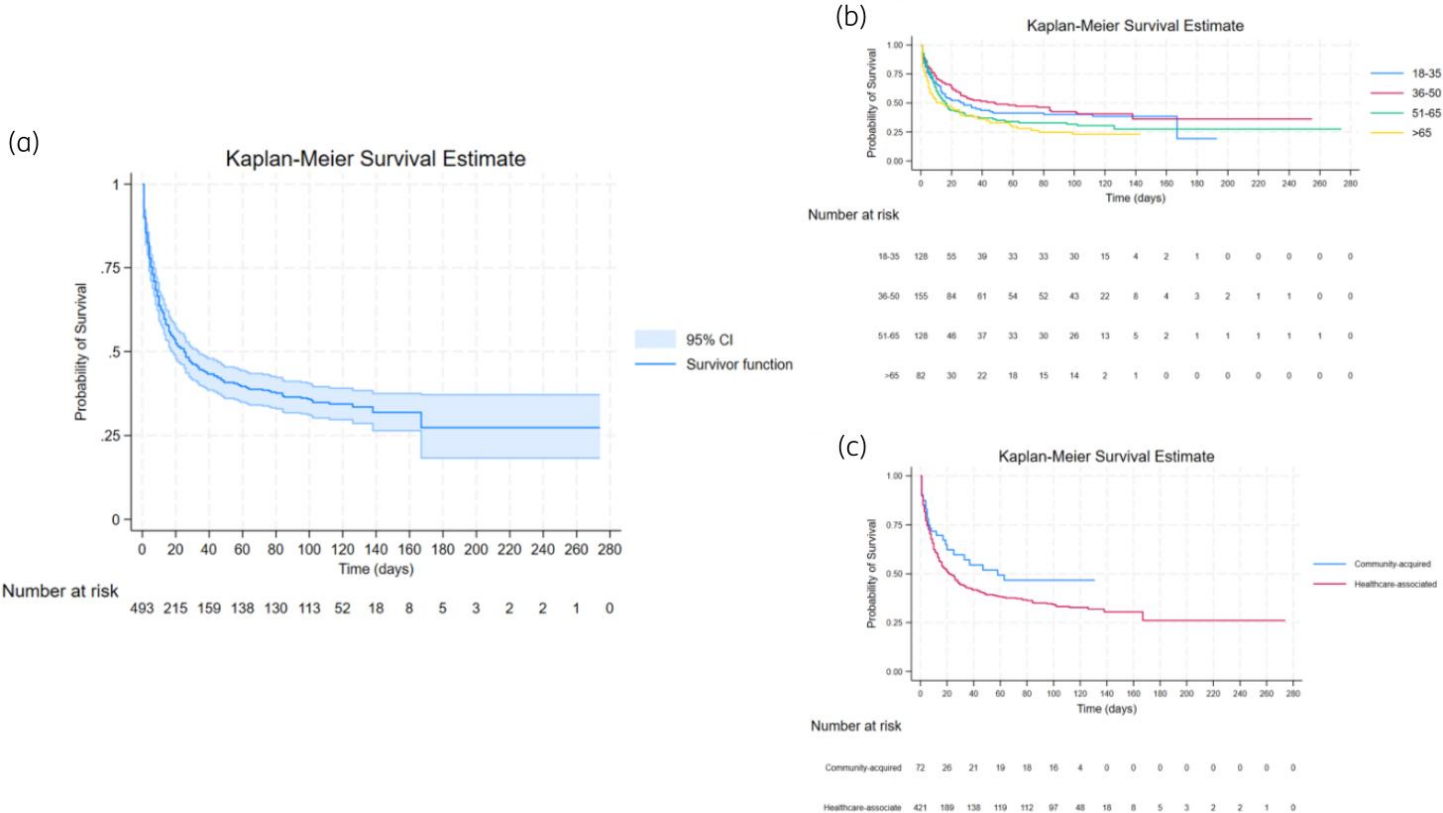
Kaplan–Meier curves for survival. (a) Kaplan–Meier survival curve of all participants. (b) Stratified by age category. (c) Stratified by community-acquired and hospital-associated infections.

The overall in-hospital CFR was 56.5% (296/524), including 10.1% (30/296) of deaths occurring on the day the sample was submitted for culture (Figure S2). The Kaplan–Meier estimation of survival for all participants is shown in Figure 2.

In the multivariate analysis, the independent risk factors for in-hospital death included urethral catheterization (aOR 3.30; 95% CI: 1.51–7.23) and an admission quick sepsis-related organ failure assessment (qSOFA) score of 1 to 3 (aOR 2.14; 95% CI: 1.25–3.68) (Table 5). Achieving source control of the *K. pneumoniae* infection was associated with lower odds of death (aOR 0.18; 95% CI: 0.10–0.30).

**Table 5.** Univariate and multivariate logistic regression analysis of variables associated with in-hospital mortality in adults with *Klebsiella pneumoniae* invasive disease

Variable^a^	Died, *n* (%) (*N* = 296)	Alive, *n* (%) (*N* = 228)	Unadjusted		Adjusted	
			OR (95% CI)	*P* value	OR (95% CI)	*P* value
Sex						
Male	153 (51.7)	136 (59.6)	Ref.	Ref.	NA	NA
Female	143 (48.3)	92 (40.4)	1.38 (0.97–1.96)	0.070	1.49 (0.88–2.54)	0.141
Age categories						
18–35 y	72 (24.3)	62 (27.2)	Ref.	Ref.	ND	ND
36–50 y	82 (27.7)	80 (35.1)	0.88 (0.56–1.40)	0.594	ND	ND
51–65 y	82 (27.7)	54 (23.7)	1.31 (0.81–2.12)	0.276	ND	ND
>65 y	60 (20.3)	32 (14.0)	1.61 (0.93–2.79)	0.086	ND	ND
Area of admission during sample collection						
ICU or high-care unit	153 (51.7)	73 (32.0)	3.34 (2.03–5.49)	<0.001	1.28 (0.59–2.77)	0.530
Medical ward	74 (25.0)	61 (26.8)	1.93 (1.13–3.30)	0.015	1.94 (0.81–4.66)	0.138
Surgical ward	37 (12.5)	59 (25.9)	Ref.	Ref.	NA	NA
Hospital						
CHBAH	174 (58.8)	126 (55.3)	Ref.	Ref.	ND	ND
CMJAH	105 (35.5)	84 (36.8)	0.91 (0.63–1.31)	0.595	ND	ND
HJH	17 (5.7)	18 (7.9)	0.68 (0.34–1.38)	0.288	ND	ND
Presence of comorbid conditions						
No	92 (31.1)	91 (39.9)	Ref.	Ref.	NA	NA
Yes	204 (68.9)	137 (60.1)	1.47 (1.03–2.11)	0.036	1.60 (0.90–2.84)	0.107
Living with HIV/AIDS						
No	213 (72.0)	170 (74.6)	Ref.	Ref.	ND	ND
Yes	83 (28.0)	58 (25.4)	1.14 (0.77–1.69)	0.506	ND	ND
Diabetes mellitus						
No	245 (82.8)	193 (84.6)	Ref.	Ref.	ND	ND
Yes	51 (17.2)	35 (15.4)	1.15 (0.72–1.84)	0.565	ND	ND
Solid tumour (non-metastatic)						
No	277 (93.6)	212 (93.0)	Ref.	Ref.	ND	ND
Yes	19 (6.4)	16 (7.0)	0.91 (0.46–1.81)	0.786	ND	ND
Moderate to severe CKD						
No	281 (94.9)	210 (92.1)	Ref.	Ref.	ND	ND
Yes	15 (5.1)	18 (7.9)	0.62 (0.31–1.26)	0.190	ND	ND
Recent surgery						
No	134 (45.3)	108 (47.4)	Ref.	Ref.	ND	ND
Yes	162 (54.7)	119 (52.2)	1.10 (0.78–1.55)	0.600	ND	ND
Presence of central venous catheter						
No	110 (37.2)	147 (64.5)	Ref.	Ref.	NA	NA
Yes	186 (62.8)	80 (35.1)	3.11 (2.17–4.45)	<0.001	1.41 (0.68–2.91)	0.356
Presence of urethral catheter						
No	90 (30.4)	137 (60.1)	Ref.	Ref.	NA	NA
Yes	205 (69.3)	89 (39.0)	3.51 (2.44–5.05)	<0.001	3.30 (1.51–7.23)	0.003
Category of infection						
Community-acquired	34 (11.5)	48 (21.1)	Ref.	Ref.	NA	NA
Healthcare-associated	262 (88.5)	180 (78.9)	2.05 (1.27–3.32)	0.003	1.43 (0.54–3.80)	0.470
Source control of infection						
Yes	48 (16.2)	121 (53.1)	0.13 (0.08–0.20)	<0.001	0.18 (0.10–0.30)	<0.001
No	199 (67.2)	64 (28.1)	Ref.	Ref.	NA	NA
qSOFA score						
0	126 (42.7)	154 (67.5)	Ref.	Ref.	NA	NA
1–3	169 (57.3)	74 (32.5)	2.79 (1.95–4.00)	<0.001	2.14 (1.25–3.68)	0.006
Delay of ≥24 h from time of culture collection until receipt of appropriate therapy^b^						
No	87 (29.4)	102 (44.7)	Ref.	Ref.	NA	NA
Yes	201 (67.9)	121 (53.1)	1.95 (1.35–2.80)	<0.001	1.19 (0.54–2.62)	0.661
Polymicrobial culture						
No	184 (62.2)	172 (75.4)	Ref.	Ref.	NA	NA
Yes	112 (37.8)	56 (24.6)	1.87 (1.28–2.74)	0.001	1.53 (0.88–2.65)	0.132
MDR						
No	91 (30.7)	109 (47.8)	Ref.	Ref.	NA	NA
Yes	205 (69.3)	119 (52.2)	2.06 (1.44–2.95)	<0.001	0.71 (0.18–2.78)	0.620
3GC resistance						
No	70 (23.6)	91 (39.9)	Ref.	Ref.	NA	NA
Yes	226 (76.4)	137 (60.1)	2.14 (1.47–3.13)	<0.001	1.34 (0.42–4.26)	0.620
CRE						
No	109 (36.8)	127 (55.7)	Ref.	Ref.	NA	NA
Yes	187 (63.2)	101 (44.3)	2.16 (1.52–3.07)	<0.001	2.39 (0.59–9.75)	0.224
OXA-48						
No	183 (61.8)	160 (70.2)	Ref.	Ref.	NA	NA
Yes	113 (38.2)	68 (29.8)	1.45 (1.01–2.10)	0.047	0.53 (0.18–1.58)	0.256
NDM						
No	286 (96.6)	221 (96.9)	Ref.	Ref.	NA	NA
Yes	10 (3.4)	7 (3.1)	1.10 (0.41–2.95)	0.844	0.61 (0.10–3.61)	0.587
OXA-48 and NDM co-detection						
No	259 (87.5)	212 (93.0)	Ref.	Ref.	NA	NA
Yes	37 (12.5)	16 (7.0)	1.89 (1.02–3.50)	0.042	0.77 (0.20–2.95)	0.707
Received appropriate antibiotic treatment^b^						
No	126 (42.6)	57 (25.0)	Ref.	Ref.	NA	NA
Yes	170 (57.4)	171 (75.0)	0.45 (0.31–0.66)	<0.001	0.74 (0.38–1.44)	0.381

CHBAH, Chris Hani Baragwanath Academic Hospital; CKD, chronic kidney disease; CMJAH, Charlotte Maxeke Johannesburg Academic Hospital; CRE, carbapenem-resistant Enterobacterales; HJH, Helen Joseph Hospital; NA, not applicable; ND, not done; NDM, New Delhi metallo-β-lactamase; OXA-48, oxacillinase-type β-lactamase; qSOFA, quick sepsis-related organ failure assessment; 3GC, third generation cephalosporin.

^a^The following variables were included a priori in the univariate analysis: area of admission during sample collection; the presence of comorbidities; recent surgery; the presence of a central venous line, the presence of a urethral catheter; category of infection; delay to receipt of appropriate therapy; polymicrobial culture; MDR; 3GC resistance, CRE; OXA-48; NDM; OXA-48 and NDM; and receipt of appropriate therapy.

^b^Appropriate antibiotic treatment was defined as the receipt of an antibiotic to which the *K. pneumoniae* was susceptible.

A successful contact of cases discharged alive from hospital was completed in 71.2% (136/191) of cases who had consented to a follow-up call, of whom 16.9% (23/136) had died, 9.6% (13/136) had been re-hospitalized and 73.5% (100/136) were considered to have recovered from the KPn-ID episode (Table 2).

## Discussion

The aims of our study were to describe the clinical and microbiological epidemiology of KPn-ID in hospitalized adults, with a focus on clinical outcomes and *K. pneumoniae* susceptibility profiles, in Johannesburg, South Africa. The principal findings in our study, undertaken in a setting with a high prevalence of HIV, included an alarming CFR (56.5%) in adults hospitalized with KPn-ID. Additionally, this was a relatively young population [median age 48 (IQR 35–61) years], and a high proportion (68.0%) had an underlying comorbidity. We also noted a high prevalence of resistance to 3GCs (69.3%) and carbapenems (55.0%), which, together with limited access to effective antimicrobials to manage MDR KPn-ID, may be contributing to the high burden of KPn-ID deaths in our setting.

Strengths of our study included a relatively large cohort of study participants, with prospective enrolment of the majority of cases over a 1 year period and a 90 day follow-up. The linkage of pertinent clinical information with microbiological data allowed for a detailed analysis of risk factors for death in KPn-ID. Limitations of our study include that cases had samples collected at the discretion of attending physicians, which could lead to an underestimation of KPn-ID burden. Also, clinical data abstraction was dependent on locating the clinical record of the patient as no electronic medical record system currently exists at any of the participating hospitals. Furthermore, the primary site of infection was determined using clinical information and supportive radiological findings recorded in the clinical records by treating clinicians. We attempted to corroborate the primary site of infection by linking temporally related *K. pneumoniae* cultures from other sample types to each *K. pneumoniae* blood or CSF culture. Other limitations in our resource-constrained setting were the intermittent shortages of rapid carbapenemase testing kits and laboratory consumables that resulted in incomplete testing for carbapenemase prevalence. Also, carbapenem and colistin MICs were not performed on all CRKp isolates. Overall, our study findings contribute to emerging data on the burden of KPn-ID in hospitalized African adults, of which there are few published studies.

A systematic review and meta-analysis of mortality from *K. pneumoniae* BSIs reported a 30 day mortality of 29.0% (95% CI: 0.26–0.31), with individual studies reporting CFRs ranging from 8.0% to 81.0% across various settings.^15^ Our reported in-hospital CFR is almost double that of the CFR reported in the systematic review and may be explained by patients having multiple comorbidities, limited access to ICU facilities, and lack of access to newer antimicrobials for treating CRKp such as ceftazidime/avibactam. Of note, 34% of in-hospital deaths in our study occurred within 72 h from culture. In comparison, a large prospective Norwegian study of *K. pneumoniae* species complex BSIs reported a cumulative CFR of ∼5% at 120 h post culture positivity.^16^

Specifically looking at CRKp, mortality rates are higher.^1,7,17–20^ We reported an in-hospital CFR of 63.2% in CRKp, which is far higher than a previous report from South Africa in 2020 of 36.6%.^17^ Studies investigating risk factors for carbapenem-resistant Enterobacterales (CRE) infections have described an association between prolonged urethral catheterization and an increased risk of CRE infection;^21,22^ however, urethral catheterization was not previously identified as an independent risk factor for 30 day mortality.^22^ In contrast, our study showed that urethral catheterization remained a significant risk factor for in-hospital death after adjusting for covariables. We also found a significant association between a qSOFA score of 1–3 and increased in-hospital CFR, providing validation for the use of qSOFA in adults with KPn-ID. The qSOFA score is a bedside tool used to identify patients with suspected infection who are at risk of a poor outcome if not managed in an ICU setting. It was calculated for all individuals enrolled in our study, based on recorded admission vital signs. Similarly, a Hong Kong study showed that a higher qSOFA score was an independent predictor of death following hospital discharge after completing treatment for KPn-ID.^23^

There is a paucity of recent studies assessing the 90 day outcomes of KPn-ID survivors. A study from 2011 reported a 72% readmission rate at 90 days in individuals previously discharged after CRKp invasive disease.^24^ Of the cases in our study with a successful 90 day contact, the readmission rate was 9.6% and CFR was 16.9%.

In our cohort, the median age of KPn-ID cases was 48 (IQR 35–61) years, compared with 62 years in a study from the USA.^19^ The lower median age of cases in our study is likely related to the high prevalence (26.9%) of HIV in our study [median age of PLWH 46 (IQR 38–54) years], which is higher than the HIV prevalence of 12.7% in the general population and 16.7% in the age category 15–49 years, respectively.^25^ Although diabetes is considered a risk factor for KPn-ID,^5,6^ 16.4% of the KPn-ID cases in our study had diabetes, which is close to the population prevalence range of 10.8%^26^ to 15.3%^27^ in South Africa. Neither HIV nor diabetes were found to be independent predictors of mortality in our study.

Most cases of KPn-ID in our study were pHAIs, in keeping with local and international surveillance studies.^5,17,19^ Furthermore, over one-third of invasive isolates were polymicrobial, with *A. baumannii* and *E. coli* identified most frequently as co-pathogens. A systematic review and meta-analysis of *A. baumannii* polymicrobial BSIs found *Klebsiella* species to account for 10% (95% CI: 6%–16%) of the co-pathogens detected.^28^ Karakonstantis *et al.*^28^ hypothesized a potential symbiotic interaction between pathogens that are frequently co-isolated, although we did not find polymicrobial infection to be an independent predictor of in-hospital death. Resistance to all β-lactam antimicrobials was high in our cohort and expectedly more common in pHAI than pCAI. South African microbiological surveillance studies have shown an increase in and predominance of HAI-related ESBL-producing *K. pneumoniae* and CRKp.^6,17,18^ In South Africa, it has been reported that certain geographical regions (Gauteng, KwaZulu Natal and the Western Cape) have a higher burden of CRE-related BSIs, with *K. pneumoniae* accounting for 79.8% of CRE-related BSIs, of which 89.5% were HAI.^17^ The prevalence of colistin-resistant isolates (7.6%) in our study is concerning considering the limited antibiotic treatment options available in low-resource settings such as ours, but is in keeping with national trends of colistin resistance prevalence.^17^ A 2022 systematic review and meta-analysis of the global prevalence of colistin resistance (based on CLSI and EUCAST criteria) in *K. pneumoniae* BSIs described a pooled global prevalence of 3.1% (95% CI: 1.5%–4.7%), with a higher prevalence (12.9%) in more recent isolates studied from 2020 compared with isolates studied between 2015 and 2019 (2.95%).^29^ Carbapenemases were detected from isolates at all study sites, with OXA-48 predominating, in keeping with local surveillance epidemiology that reflects the predominance of OXA-48 and NDM carbapenemases.^17,30^ Of concern is the presence of CRKp isolates with OXA-48 and NDM carbapenemases co-detected, as the management of these patients requires antimicrobials that are largely unavailable in the public sector in South Africa.^4,30^

Important unanswered questions arose from our study findings and warrant further investigation. The significant association between the presence of a urethral catheter and in-hospital mortality requires intensified infection prevention and control audits at all study sites and targeted measures in order to prevent *K. pneumoniae* infections. Although it is not known whether the readmissions or out-of-hospital deaths were related to the initial KPn-ID episode, the morbidity and mortality outcomes following discharge after an episode of KPn-ID warrant further investigation.

In conclusion, our study described the clinical outcomes and *K. pneumoniae* susceptibility profiles of adults with KPn-ID hospitalized in Johannesburg, South Africa. Our study highlights the significant in-hospital mortality associated with KPn-ID in Johannesburg, South Africa. The burden of KPn-ID in our setting is largely attributable to pHAIs and CRKp, highlighting the need for rapid identification and susceptibility testing of the organism, and prompt initiation of appropriate treatment. Early source control of the *K. pneumoniae* infection is necessary in the management of patients.

## Supplementary Material

dlag003_Supplementary_Data

## Data sharing

The data used in this study are not publicly available.

## Supplementary data

Figures S1 and S2 and Tables S1 to S6 are available as Supplementary data at *JAC-AMR* Online.

## References

[1] Murray CJL, Ikuta KS, Sharara F et al Global burden of bacterial antimicrobial resistance in 2019: a systematic analysis. Lancet 2022; 399: 629–55. 10.1016/S0140-6736(21)02724-0 35065702 PMC8841637

[2] Chang D, Sharma L, Dela Cruz CS et al Clinical epidemiology, risk factors, and control strategies of *Klebsiella pneumoniae* infection. Front Microbiol 2021; 12: 750662. 10.3389/fmicb.2021.750662 34992583 PMC8724557

[3] Sartorius B, Gray AP, Weaver ND et al The burden of bacterial antimicrobial resistance in the WHO African region in 2019: a cross-country systematic analysis. Lancet Glob Health 2024; 12: e201–16. 10.1016/S2214-109X(23)00539-9 38134946 PMC10805005

[4] Finlayson H, Chibabhai V, Jeena P et al The changing landscape of antimicrobial resistance and use in South Africa: the need for access to new antibiotics: a position paper. S Afr Med J 2024; 114: e2348. https://scielo.org.za/pdf/samj/v114n10/09.pdf. 39508224 10.7196/SAMJ.2024.v114i10.2348

[5] Wyres KL, Lam MMC, Holt KE. Population genomics of *Klebsiella pneumoniae*. Nat Rev Microbiol 2020; 18: 344–59. 10.1038/s41579-019-0315-1 32055025

[6] Perovic O, Ismail H, Quan V et al Carbapenem-resistant Enterobacteriaceae in patients with bacteraemia at tertiary hospitals in South Africa, 2015 to 2018. Eur J Clin Microbiol Infect Dis 2020; 39: 1287–94. 10.1007/s10096-020-03845-4 32124106

[7] Brink A . Epidemiology of carbapenem-resistant Gram-negative infections globally. Curr Opin Infect Dis 2019; 32: 609–16. 10.1097/QCO.0000000000000608 31567571

[8] Statistics South Africa. Census 2022. 2023. https://census.statssa.gov.za/#/

[9] Chris Hani Baragwanath Hospital. General information. https://www.chrishanibaragwanathhospital.co.za/

[10] Charlotte Maxeke Johannesburg Academic Hospital. Wikipedia entry. https://en.wikipedia.org/wiki/Charlotte_Maxeke_Johannesburg_Academic_Hospital

[11] South African Doctors. Helen Joseph Hospital general information. https://doctors-hospitals-medical-cape-town-south-africa.blaauwberg.net/hospitals_clinics_state_hospitals/state_public_hospitals_clinics_gauteng_south_africa/helen_joseph_hospital_auckland_park_johannesburg_gauteng_south_africa

[12] CLSI . Performance Standards for Antimicrobial Susceptibility Testing–Thirty-Third Edition: M100. 2023.

[13] CLSI . Performance Standards for Antimicrobial Susceptibility Testing–Thirty-Fourth Edition: M100. 2024.

[14] Magiorakos AP, Srinivasan A, Carey RB et al Multidrug-resistant, extensively drug-resistant and pandrug-resistant bacteria: an international expert proposal for interim standard definitions for acquired resistance. Clin Microbiol Infect 2012; 18: 268–81. 10.1111/j.1469-0691.2011.03570.x 21793988

[15] Li D, Huang X, Rao H et al *Klebsiella pneumoniae* bacteremia mortality: a systematic review and meta-analysis. Front Cell Infect Microbiol 2023; 13: 1157010. 10.3389/fcimb.2023.1157010 37153146 PMC10159367

[16] Fostervold A, Raffelsberger N, Heltland M et al Risk of death in *Klebsiella pneumoniae* bloodstream infections is associated with specific phylogenetic lineages. J Infect 2024; 88: 106155. 10.1016/j.jinf.2024.106155 38574775

[17] Lowe M, Shuping L, Perovic O. Carbapenem-resistant Enterobacterales in patients with bacteraemia at tertiary academic hospitals in South Africa, 2019–2020: an update. S Afr Med J 2022; 112: 542–52. 10.7196/SAMJ.2022.v112i8.16351 36214398

[18] Tootla HD, Prentice E, Moodley C et al Carbapenem-resistant Enterobacterales among hospitalized patients in Cape Town, South Africa: clinical and microbiological epidemiology. JAC Antimicrob Resist 2024; 6: dlae051. 10.1093/jacamr/dlae051 38523732 PMC10959510

[19] Roach DJ, Sridhar S, Oliver E et al Clinical and genomic characterization of a cohort of patients with *Klebsiella pneumoniae* bloodstream infection. Clin Infect Dis 2024; 78: 31–9. 10.1093/cid/ciad507 37633257 PMC10810715

[20] Wang M, Earley M, Chen L et al Clinical outcomes and bacterial characteristics of carbapenem-resistant *Klebsiella pneumoniae* complex among patients from different global regions (CRACKLE-2): a prospective, multicentre, cohort study. Lancet Infect Dis 2022; 22: 401–12. 10.1016/S1473-3099(21)00399-6 34767753 PMC8882129

[21] Sharma K, Tak V, Nag VL et al An observational study on carbapenem-resistant Enterobacterales (CRE) colonisation and subsequent risk of infection in an adult intensive care unit (ICU) at a tertiary care hospital in India. Infect Prev Pract 2023; 5: 100312. 10.1016/j.infpip.2023.100312 37868258 PMC10585280

[22] Li X, Ye H. Clinical and mortality risk factors in bloodstream infections with carbapenem-resistant Enterobacteriaceae. Can J Infect Dis Med Microbiol 2017; 2017: 6212910. 10.1155/2017/6212910 29379527 PMC5742906

[23] Man MY, Shum HP, Li KC et al Impact of appropriate empirical antibiotics on clinical outcomes in *Klebsiella pneumoniae* bacteraemia. Hong Kong Med J 2021; 27: 247–57. 10.12809/hkmj208698 34393109

[24] Neuner E, Yeh J, Hall G et al Treatment and outcomes in carbapenem-resistant *Klebsiella pneumoniae* bloodstream infections. Diagn Microbiol Infect Dis 2011; 69: 357–62. 10.1016/j.diagmicrobio.2010.10.013 21396529 PMC3058153

[25] Statistics South Africa. Mid-year population estimates 2024. 2024. https://www.statssa.gov.za/publications/P0302/P03022024.pdf

[26] World Bank Group. Diabetes prevalence (% of population ages 20 to 70)—South Africa. 2021. https://data.worldbank.org/indicator/SH.STA.DIAB.ZS?locations=ZA

[27] Pheiffer C, Pillay-van Wyk V, Turawa E et al Prevalence of type 2 diabetes in South Africa: a systematic review and meta-analysis. Int J Environ Res Public Health 2021; 18: 5868. 10.3390/ijerph18115868 34070714 PMC8199430

[28] Karakonstantis S, Ioannou P, Kritsotakis E. Co-isolates of *Acinetobacter baumannii* complex in polymicrobial infections: a meta-analysis. Access Microbiol 2022; 4: acmi000348. 10.1099/acmi.0.000348 36003364 PMC9394532

[29] Uzairue LI, Rabaan AA, Adewumi FA et al Global prevalence of colistin resistance in *Klebsiella pneumoniae* from bloodstream infection: a systematic review and meta-analysis. Pathogens 2022; 11: 1092. 10.3390/pathogens11101092 36297149 PMC9607870

[30] Marais G, Moodley C, Claassen-Weitz S et al Carbapenem-resistant *Klebsiella pneumoniae* among hospitalized patients in Cape Town, South Africa: molecular epidemiology and characterization. JAC Antimicrob Resist 2024; 6: dlae050. 10.1093/jacamr/dlae050 38529003 PMC10963078

