# Supplementary material for: Clinical and microbiological epidemiology of Klebsiella pneumoniae invasive disease in hospitalized adults in Johannesburg, South Africa: a multicentre observational study
Source: JAC Antimicrob Resist. 2026 Jan 29;8(1):dlag003. doi: 10.1093/jacamr/dlag003 (PMC12852996; doi:10.1093/jacamr/dlag003)
Supplement: dlag003_Supplementary_Data [file dlag003_supplementary_data.docx]

**SUPPLEMENTARY MATERIALS**

**Table S1. Microbiological characteristics of *K. pneumoniae* invasive disease in adults (Enrolled with hospital breakdown and unenrolled)**

| **Variable** |  | | **Patients, No. (%)^a^** | | | |
| --- | --- | --- | --- | --- | --- | --- |
|  | **Enrolled cases**  **(n= 524)** | | | | **Total**  **Enrolled cases**  **(N= 524)** | **Total**  **Unenrolled cases**  **(N = 93)** |
|  | **CHBAH**  **(n= 300)** | **CMJAH**  **(n= 189)** | | **HJH**  **(n= 35)** |  |  |
| Sample type  Blood culture  CSF | 294 (98.0)  6 (2.0) | 189 (100.0)  0 | | 35 (100.0) 0 | 518 (98.9)  6 (1.1) | 91 (97.8)  2 (2.2) |
| Polymicrobial bacteraemia | 107 (35.7) | 48 (25.4) | | 13 (37.1) | 168 (32.1) | 32 (34.4) |
| Resistance^b^  Amoxicillin-clavulanic acid  Ciprofloxacin  Ceftriaxone  Ceftazidime  Cefepime  Gentamicin  Amikacin  Piperacillin-tazobactam  Ertapenem Resistant  MIC ≥ 2 mg/L  No MIC result reported  Ertapenem Intermediate  MIC ≥ 1 and < 2 mg/L  No MIC result reported  Imipenem Resistant  MIC ≥ 4 mg/L  No MIC result reported  Imipenem Intermediate  MIC ≥ 2 and < 4 mg/L  No MIC result reported  Meropenem Resistant  MIC ≥ 4 mg/L  No MIC result reported  Meropenem Intermediate  MIC ≥ 2 and < 4 mg/L  No MIC result reported  Tigecycline  Colistin  MIC ≤ 2 mg/L  MIC ≥ 4 mg/L  Ceftazidime-avibactam | 211/297 (71.0)  166/287 (57.8)  205/300 (68.3)  202/298 (67.8)  202/300 (67.3)  135/299 (45.2)  98/300 (32.7)  189/300 (63.0)  167/298 (56.0)  136/298 (45.6)  31/298 (10.4)  3/298 (1.0)  3/298 (1.0)  0/298 (0)  134/298 (45.0)  106/298 (35.6)  28/298 (9.4)  20/298 (6.7)  16/298 (5.4)  4/298 (1.3)  141/297 (47.5)  116/297 (39.1)  25/297 (8.4)  16/297 (5.4)  15/297 (5.1)  1/297 (0.3)  4/26 (15.4)  152/157 (96.8)  5/157 (3.2)  0 | 137/189 (72.5)  120/189 (63.5)  141/189 (74.6)  134/188 (71.3)  124/189 (65.6)  93/188 (49.5)  71/189 (37.6)  122/187 (65.2)  84/187 (44.9)  76/187 (40.6)  8/187 (4.3)  3/187 (1.6)  3/187 (1.6)  0/187 (0)  67/189 (35.4)  59/189 (31.2)  8/189 (4.2)  8/189 (4.2)  7/189 (3.7)  1/189 (0.5)  72/189 (38.1)  65/189 (34.4)  7/189 (3.7)  6/189 (3.2)  6/189 (3.2)  0/189 (0)  18/178 (10.1)  78/92 (84.8)  14/92 (15.2)  1/4 (25.0) | | 14/35 (40.0)  13/34 (38.2)  17/35 (48.6)  14/35 (40.0)  9/35 (25.7)  11/34 (32.4)  4/34 (11.8)  13/35 (37.1)  8/35 (22.9)  7/35 (20.0)  1/35 (2.9)  0/35 (0)  0/35 (0)  0/35 (0)  7/35 (20.0)  7/35 (20.0)  0/35 (0)  0/35 (0)  0/35 (0)  0/35 (0)  7/35 (20.0)  6/35 (17.1)  1/35 (2.9)  0/35 (0)  0/35 (0)  0/35 (0)  2/19 (10.5)  2/2 (100.0)  0/2 (0)  0 | 362/521 (69.5)  299/510 (58.6)  363/524 (69.3)  350/521 (67.2)  335/524 (63.9)  239/521 (45.9)  173/523 (33.1)  324/522 (62.1)  259/520 (49.8)  219/520 (42.1)  40/520 (7.7)  6/520 (1.2)  6/520 (1.2)  0/520 (0)  208/522 (39.8)  172/522 (33.0)  36/522 (6.9)  28/522 (5.4)  23/522 (4.4)  5/522 (1.0)  220/521 (42.2)  187/521 (35.9)  33/521 (6.3)  22/521 (4.2)  21/521 (4.0)  1/521 (0.2)  24/223 (10.8)  232/251 (92.4)  19/251 (7.6)  1/4 (25.0) | 61/93 (65.6)  58/91 (63.7)  60/92 (65.2)  60/93 (64.5)  58/93 (62.4)  41/93 (44.1)  24/93 (25.8)  55/92 (59.8)  44/93 (47.3)  20/93 (21.5)  24/93 (25.8)  0/93 (0)  0/93 (0)  0/93 (0)  30/93 (32.3)  15/93 (16.1)  15/93 (16.1)  8/93 (8.6)  4/93 (4.3)  4/93 (4.3)  31/93 (33.3)  17/93 (18.3)  14/93 (15.1)  8/93 (8.6)  5/93 (5.4)  3/93 (3.2)  5/41 (12.2)  29/31 (93.5)  2/31 (6.5)  0 |
| Resistance pattern  3GC  CRKp  MDR | N= 300  205 (68.3)  175 (58.3)  191 (63.7) | N= 189  141 (74.6)  105 (55.6)  120 (63.5) | | N= 35  17 (48.6)  8 (22.9)  13 (37.1) | N= 524  363 (69.3)  288 (55.0)  324 (61.8) | N= 93  60 (65.2)  47 (50.5)  56 (60.2) |
| Carbapenemase tested  OXA-48  NDM  OXA-48 and NDM  NDM and VIM  No carbapenemase detected | N= 150  130 (86.7)  12 (8.0)  6 (4.0)  0  2 | N= 97  49 (50.5)  4 (4.1)  44 (45.4)  0  0 | | N= 6  2 (33.3)  1 (16.7)  3 (50.0)  0  0 | N= 253  181 (71.5)  17 (6.7)  53 (20.9)  0  2 | N= 40  27 (67.5)  5 (12.5)  7 (17.5)  1 (2.5)  0 |

Abbreviations: CHBAH, Chris Hani Baragwanath Academic Hospital; CMJAH, Charlotte Maxeke Johannesburg Academic Hospital; HJH, Helen Joseph Hospital; CR, complete resistance; IR, intermediate resistance; 3GC, 3^rd^ generation cephalosporin; CRKp, carbapenem-resistant *K. pneumoniae*; OXA-48, oxacillinase-type β-lactamase; NDM, New Delhi metallo-β-lactamase; VIM, Verona integron-encoded metallo-β-lactamase.

^a^ Data represent no. (%) of patients unless otherwise specified

^b^ Resistance prevalence includes only isolates that are fully resistant. Intermediate resistance prevalence and stratification according to MIC is included for carbapenems only

**Table S2. Polymicrobial bacteraemia organism breakdown**

|  | **CHBAH**  **(n= 300)** | **CMJAH**  **(n= 189)** | **HJH**  **(n= 35)** | **Not enrolled**  **(n= 93)** |
| --- | --- | --- | --- | --- |
| Polymicrobial bacteraemia | 107 (35.7) | 48 (25.4) | 13 (37.1) | 32 (34.4) |
| Number of times isolated  *Acinetobacter baumannii*  *Escherichia coli*  *Enterococcus faecalis*  *Enterococcus faecium*  *Pseudomonas aeruginosa*  *Enterobacter species*  *Proteus species*  *Citrobacter species*  *Staphylococcus aureus*  *Serratia marcescens*  *Salmonella enterica*  *Providencia species*  *Klebsiella oxytoca*  *Stenotrophomonas maltophilia*  *Streptococcus pneumoniae*  *Viridans Streptococcus*  Group B *Streptococcus*  Group D *Streptococcus*  Group A *Streptococcus*  Coagulase negative Staphylococcus/ Skin colonisers | 38  24  23  13  11  11  11  5  5  1  0  1  0  0  1  1  0  0  2  7 | 6  10  7  8  3  5  6  1  1  2  0  1  1  1  0  0  0  1  0  3 | 2  1  1  0  1  1  0  1  3  0  1  0  0  0  0  1  0  0  0  2 | 6  6  6  6  1  4  0  1  1  0  0  0  0  2  0  0  1  0  0  4 |

Abbreviations: CHBAH, Chris Hani Baragwanath Academic Hospital; CMJAH, Charlotte Maxeke Johannesburg Academic Hospital; HJH, Helen Joseph Hospital

**Table S3. Opportunistic Infections in Adults with *K. pneumoniae* invasive disease**

| **Variable** | **Patients, No. (%)^a^** |
| --- | --- |
| Opportunistic Infections in PLWH (n= 141)  *Pneumocystis jirovecii* pneumonia  Cryptococcal meningitis  *Mycobacterium tuberculosis* infection^b^ | 2 (1.4)  6 (4.3)  25 (17.7) |
| *Mycobacterium tuberculosis* infection in HIV negative^c^ (n= 234) | 7 (3.0) |

^a^ Data represent no. (%) of patients unless otherwise specified

^b^ Pulmonary tuberculosis= 13; abdominal tuberculosis= 8; tuberculous meningitis= 3; genitourinary tuberculosis= 1

^c^ Pulmonary tuberculosis= 4; abdominal tuberculosis= 2; spinal tuberculosis= 1

**Table S4. Non-benign comorbidities in Adults with *K. pneumoniae* invasive disease**

| **Non-benign comorbidity** | **Patients,**  **N= 524 (%)^a^** | **Males**  **n= 289 (55.2%)** | **Females**  **n= 235 (44.8%)** |
| --- | --- | --- | --- |
| Non-metastatic cancer  Liver/ gallbladder/ bile ducts  Brain  Prostate  Cervix  Pancreas  Bladder  Colorectal  Thyroid  Ovary  Stomach | 35 (6.7)  8/35 (22.9)  6/35 (17.1)  5/35 (14.3)  4/35 (11.4)  3/35 (8.6)  3/35 (8.6)  2/35 (5.7)  2/35 (5.7)  1/35 (2.9)  1/35 (2.9) | 21 (7.3)  5/21 (23.8)  5/21 (23.8)  5/21 (23.8)  0  3/21 (14.3)  2/21 (9.5)  1/21 (4.8)  0  0  0 | 14 (6.0)  3/14 (21.4)  1/14 (7.1)  0  4/14 (28.6)  0  1/14 (7.1)  1/14 (7.1)  2/14 (14.3)  1/14 (7.1)  1/14 (7.1) |
| Metastatic cancer  Pancreas  Liver/ gallbladder/ bile ducts  Breast  Prostate  Colorectal  Bladder  Testicular  Oesophagus  Kidney  Thyroid  Neuroendocrine | 25 (4.8)  6/25 (24.0)  4/25 (16.0)  3/25 (12.0)  3/25 (12.0)  3/25 (12.0)  1/25 (4.0)  1/25 (4.0)  1/25 (4.0)  1/25 (4.0)  1/25 (4.0)  1/25 (4.0) | 14 (4.8)  3/14 (21.4)  1/14 (7.1)  0  3/14 (21.4)  2/14 (14.3)  1/14 (7.1)  1/14 (7.1)  1/14 (7.1)  1/14 (7.1)  0  1/14 (7.1) | 11 (4.7)  3/11 (27.3)  3/11 (27.3)  3/11 (27.3)  0  1/11 (9.1)  0  0  0  0  1/11 (9.1)  0 |
| Leukaemia  Acute myeloid leukaemia  Acute promyelocytic leukaemia  Acute lymphoblastic leukaemia  Chronic lymphocytic leukaemia | 21 (4.0)  9/21 (42.9)  6/21 (28.6)  3/21 (14.3)  3/21 (14.3) | 10 (3.5)  4/10 (40.0)  3/10 (30.0)  1/10 (10.0)  2/10 (20.0) | 11 (4.7)  5/11 (45.5)  3/11 (27.3)  2/11 (18.2)  1/11 (9.1) |
| Lymphoma  Hodgkin’s Lymphoma  Non-Hodgkin’s Lymphoma  Diffuse large B-cell  Follicular  Undifferentiated  Other (not specified)  Multiple Myeloma | 17 (3.2)  4/17 (23.5)  6/17 (35.3)  1/17 (5.9)  1/17 (5.9)  2/17 (11.8)  3/17 (17.6) | 11 (3.8)  2/11 (18.2)  5/11 (45.5)  1/11 (9.1)  0  1/11 (9.1)  2/11 (18.2) | 6 (2.6)  2/6 (33.3)  1/6 (16.7)  0  1/6 (16.7)  1/6 (16.7)  1/6 (16.7) |

^a^ Data represent no. (%) of patients unless otherwise specified

**Table S5. Surgical procedures/ interventions**

| **Type** | **Specific surgery** | **N (%)**  **N= 281** |
| --- | --- | --- |
| Laparotomy | Exploratory laparotomy n= 82  Bowel resection n= 13  Hernia repair n= 3  Stoma reversal n= 5 | 103 |
| Laparoscopic | Cholecystectomy n= 1 | 1 |
| Interventional radiology | Angioembolisation n= 2  Balloon dilatation n= 1  Percutaneous biliary drainage n= 5  Pigtail drain n= 3 | 11 |
| Airway | Awake intubation n= 2  Tracheostomy n= 16 | 18 |
| Endoscopy and biopsy | Gastroscopy n= 8  Percutaneous endoscopic gastrostomy n= 1 | 9 |
| Hepato-pancreato-biliary | Endoscopic retrograde cholangiopancreatography n= 18  Common bile duct stent n= 1  Liver biopsy n= 1 | 20 |
| Urological | Cystoscopy n= 2  Ureteric stent insertion or removal n= 3  Prostate biopsy n= 2  Suprapubic catheter insertion n= 1  Radical cystectomy n= 2  Radical orchidectomy n= 1  Prostatectomy n= 1 | 12 |
| Renal Dialysis related | Quinton line insertion or exchange n= 3  Tenkoff line removal n= 1 | 4 |
| Obstetrics and Gynaecology | Caesarean section n= 2  Hysterotomy n= 1  Total abdominal hysterectomy n= 1  Uterine evacuation n= 3 | 7 |
| Orthopaedic | Above knee amputation n= 3  Below knee amputation n= 3  Ray amputation n= 1  Open reduction and internal fixation n= 1  External fixation device n= 1  Intramedullary nail n= 3  Spinal surgery n= 2 | 14 |
| Skin and soft tissue debridement Incision and drainage of abscess  Biopsy | Debridement n= 37  Incision and drainage of abscess n= 2  Biopsy soft tissue n= 1 | 40 |
| Solid organ transplant | Liver n= 1  Renal n= 4 | 5 |
| Cardiac | Lobectomy n= 1  Thoracotomy n= 1  Cardiac valve replacement n= 3  Removal septic pacemaker n= 1  Thoracic Endovascular Aortic Repair n= 1 | 7 |
| Neurosurgical | Burr holes n= 2  Craniotomy n= 8  External ventricular drain insertion or removal n= 3 | 13 |
| Vascular surgery | Neck exploration n= 1  Repair abdominal aortic aneurysm n= 1 | 2 |
| Endocrine surgery | Parathyroidectomy n= 1  Thyroidectomy n= 4 | 5 |
| Chemotherapy | Chemotherapy n= 9  Stem cell transplant n= 1 | 10 |

**Table S6. Inappropriate antimicrobial treatment in adults with *K. pneumoniae* invasive disease**

| **Variable** | **Overall,**  **n (%)^a^**  **(N= 179)** | **3GC Susceptible**  **(N= 18)** | **3GC Resistant**  **(N= 161)** | **CRKp**  **(N= 144)** | **MDR**  **(N= 153)** |
| --- | --- | --- | --- | --- | --- |
| Did not receive any antibiotics^b^  Died on day of culture before antibiotic  Decision not to treat (suspected colonisation)  Reason unknown  Antibiotic not in stock (not given)  Decision not to treat (poor prognosis) | N= 13  7 (53.8)  2 (15.4)  2 (15.4)  1 (7.7)  1 (7.7) | N= 8  4 (50.0)  2 (25.0)  2 (25.0)  0  0 | N= 5  3 (60.0)  0  0  1 (20.0)  1 (20.0) | N= 2  1 (50.0)  0  0  0  1 (50.0) | N= 3  1 (33.3)  0  0  1 (33.3)  1 (33.3) |
| Did not receive targeted therapy^c^  Failure to act on result^d^  Died on day of culture before result available  Reason unknown  Decision not to treat (poor prognosis)  Culture result pending  Decision not to treat (suspected colonisation)  Alternative/ salvage therapy  Amikacin monotherapy  Carbapenem IR monotherapy  High dose meropenem infusion  Decision not to treat (clinically stable)  Antibiotic not in stock (application pending)  Patient declined admission/ treatment | N= 166  63 (38.0)  43 (25.9)  13 (7.8)  11 (6.6)  8 (4.8)  7 (4.2)  6 (3.6)  5 (3.0)  3 (1.8)  5 (3.0)  1 (0.6)  1 (0.6) | N= 10  2 (20.0)  5 (50.0)  2 (20.0)  1 (10.0)  0  0  0  0  0  0  0  0 | N= 156  61 (39.1)  38 (24.4)  11 (7.1)  10 (6.4)  8 (5.1)  7 (4.5)  6 (3.8)  5 (3.2)  3 (1.9)  5 (3.2)  1 (0.6)  1 (0.6) | N= 142  54 (38.0)  32 (22.5)  11 (7.7)  11 (7.7)  7 (4.9)  6 (4.2)  6 (4.2)  5 (3.5)  3 (2.1)  5 (3.5)  1 (0.7)  1 (0.7) | N= 150  56 (37.3)  36 (24.0)  11 (7.3)  11 (7.3)  8 (5.3)  7 (4.7)  6 (4.0)  5 (3.3)  3 (2.0)  5 (3.3)  1 (0.7)  1 (0.7) |

Abbreviations: 3GC, third generation cephalosporin; CRKp, carbapenem-resistant *K. pneumoniae*; IR, intermediate resistance; CHBAH, Chris Hani Baragwanath Academic Hospital; CMJAH, Charlotte Maxeke Johannesburg Academic Hospital; HJH, Helen Joseph Hospital

^a^ Data represent no. (%) of patients unless otherwise specified

^b^ Patients that did not receive antibiotics (N=13): CHBAH n= 9/300; CMJAH n= 3/189; HJH n=1/35

^c^ Patients that did not receive targeted therapy (N=166): CHBAH n=121/300; CMJAH n=36/189; HJH n=9/35

^d^ Uncertain if clinicians aware of culture result

**Figure S1. Antibiotics received in adults with *K. pneumoniae* invasive disease**

Abbreviations: pCAI, presumed community-associated; pHAI, presumed healthcare-associated.

**Figure S2. Cumulative deaths (%) by time from *K. pneumoniae* culture**
